# Rapid evolution of distinct *Helicobacter pylori* subpopulations in the Americas

**DOI:** 10.1371/journal.pgen.1006546

**Published:** 2017-02-23

**Authors:** Kaisa Thorell, Koji Yahara, Elvire Berthenet, Daniel J. Lawson, Jane Mikhail, Ikuko Kato, Alfonso Mendez, Cosmeri Rizzato, María Mercedes Bravo, Rumiko Suzuki, Yoshio Yamaoka, Javier Torres, Samuel K. Sheppard, Daniel Falush

**Affiliations:** 1 Microbiology, Tumour and Cell Biology, Karolinska Institutet, Stockholm, Sweden; 2 Dept. of Bacteriology II, National Institute of Infectious Diseases, Tokyo, Japan; 3 Medical Microbiology and Infectious Disease group, Swansea University, Swansea, Wales, United Kingdom; 4 Integrative Epidemiology Unit, School of Social and Community Medicine, University of Bristol, Bristol, United Kingdom; 5 Karmanos Cancer Institute, Wayne State University, Detroit, Michigan, United States of America; 6 Instituto Politecnico Nacional, ENCB, Mexico City, Mexico; 7 Dipartimento di Ricerca Traslazionale e Nuove Tecnologie in Medicina e Chirurgia, Universitá di Pisa, Pisa, Italy; 8 Grupo de Investigación en Biología del Cáncer, Instituto Nacional de Cancerología, Bogota, Colombia; 9 Dept. of Environmental and Preventive Medicine, Oita University Faculty of Medicine, Oita, Japan; 10 Unidad de Investigación en Enfermedades Infecciosas, UMAE Pediatria, IMSS, Mexico City, Mexico; 11 Milner Center for Evolution, Dept. of Biology and Biochemistry, University of Bath, Bath, United Kingdom; University of California Davis, UNITED STATES

## Abstract

For the last 500 years, the Americas have been a melting pot both for genetically diverse humans and for the pathogenic and commensal organisms associated with them. One such organism is the stomach-dwelling bacterium *Helicobacter pylori*, which is highly prevalent in Latin America where it is a major current public health challenge because of its strong association with gastric cancer. By analyzing the genome sequence of *H*. *pylori* isolated in North, Central and South America, we found evidence for admixture between *H*. *pylori* of European and African origin throughout the Americas, without substantial input from pre-Columbian (hspAmerind) bacteria. In the US, strains of African and European origin have remained genetically distinct, while in Colombia and Nicaragua, bottlenecks and rampant genetic exchange amongst isolates have led to the formation of national gene pools. We found three outer membrane proteins with atypical levels of Asian ancestry in American strains, as well as alleles that were nearly fixed specifically in South American isolates, suggesting a role for the ethnic makeup of hosts in the colonization of incoming strains. Our results show that new *H*. *pylori* subpopulations can rapidly arise, spread and adapt during times of demographic flux, and suggest that differences in transmission ecology between high and low prevalence areas may substantially affect the composition of bacterial populations.

## Author summary

*Helicobacter pylori* is one of the best studied examples of an intimate association between bacteria and humans, due to its ability to colonize the stomach for decades and to transmit from generation to generation. A number of studies have sought to link diversity in *H*. *pylori* to human migrations but there are some discordant signals such as an “out of Africa” dispersal within the last few thousand years that has left a much stronger signal in bacterial genomes than in human ones. In order to understand how such discrepancies arise, we have investigated the evolution of *H*. *pylori* during the recent colonization of the Americas. We find that bacterial populations evolve quickly and can spread rapidly to people of different ethnicities. Distinct new bacterial subpopulations have formed in Colombia from a European source and in Nicaragua and the US from African sources. Genetic exchange between bacterial populations is rampant within Central and South America but is uncommon within North America, which may reflect differences in prevalence. Our results also suggest that adaptation of bacteria to particular human ethnic groups may be confined to a handful of genes involved in interaction with the immune system.

## Introduction

In 1492, Christopher Columbus initiated a rapid colonization of the New World, principally by European migrants and Africans brought as slaves that had catastrophic consequences for the indigenous population. The new migrants brought unfamiliar weapons and pathogens [1], including new populations of the stomach-colonizing bacterium *Helicobacter pylori*. *H*. *pylori* can persist for decades in the stomach, and is often transmitted vertically from parent to child but can also be acquired from individuals in close proximity. *H*. *pylori* evolves rapidly by both mutation and homologous recombination with other co-colonizing strains [2].

Studies of the global diversity of *H*. *pylori* have shown that Europeans, Africans and Native Americans carry genetically distinct populations of bacteria; named hpEurope, hpAfrica1 and hpAfrica2, and hspAmerind, respectively [3]. The relationships between bacterial populations reflect differentiation that has arisen during the complex migration history of humans, with the prefix “hp” indicating a population and “hsp” indicating a subpopulation, which are genetically distinct from each other but less differentiated than populations. hspAmerind bacteria are presumed to be descendants of the strains present in the Americas prior to 1492, and are a subpopulation of hspEAsia, which is found in Asian countries such as China and Japan. However, these strains are rare even within groups with substantial Native American ancestry and may being dying out in competition with other strains, due to low diversity within the population or other factors [4]. hpEurope bacteria are themselves ancient hybrids between two populations, whose close relatives are currently found in unadmixed populations in North East Africa (hpNEAfrica) and central Asia (hpAsia2). The Tyrolean Iceman, Ötzi, who died 5300 years ago in central Europe, was infected by an hpAsia2 strain with little or no African ancestry [5], suggesting that the admixture probably took place within the last few thousand years.

In Latin America, gastric cancer is a leading cause of cancer death, and some countries in the region have among the highest mortality rates worldwide [6]. However, the mortality rates vary in different geographic regions, both between neighboring countries and within nations [6,7]. Several studies have been performed comparing *H*. *pylori* ancestry in high- and low risk areas and have linked phylogeographic origin of the bacteria, as well as discordant origin of bacteria and host, to increased risk of gastric cancer development [3,8]. However, these studies have been performed using MLST analysis that, being based only on seven housekeeping genes, is limited in its resolution compared to whole-genome comparisons.

To investigate if American *H*. *pylori* strains have differentiated from those found in the Old World by mixture, genetic drift or natural selection, we combined hundreds of publicly available genomes with over hundred newly sequenced genomes of *H*. *pylori* sampled in Latin America (Mexico, Nicaragua, and Colombia), Europe, and Central Asia. We show that the American bacterial populations have undergone substantial evolution within 500 years and our results also suggest that *H*. *pylori* transmission biology has been as important as human migration in determining extant patterns of diversity.

## Results

We used the Chromopainter/fineSTRUCTURE pipeline [9,10] to assess the population structure within our global collection of isolates (n = 401, described in S1 Table). Insight into the ancestry of each isolate is obtained by treating it as a “recipient” and using Chromopainter to fit it as a mosaic of DNA chunks, i.e. sets of contiguous SNPs, from a “donor panel” of other genomes. The painting can be interpreted genealogically as described in more detail in [9], namely in the local genealogy for any of the sites within a given chunk, the most recent coalescence involving the recipient individual is with the donor individual for that chunk. Each chunk thus provides information on the most recent clonal relationships and/or genetic exchange between different strains in the sample. In *H*. *pylori* recombination rates are very high and unless individuals in close proximity are sampled, it is rare to find clear evidence of direct clonal descent [11].

We used two different donor panels. A first consisted only of Old World (European, African and Asian) isolates. Since we expect that almost all of the gene flow historically has been from the Old World to the New World, using an Old World panel allows us to investigate the origins of each New World *H*. *pylori*, without the complication of determining how the strains are related to each other. Although we are principally interested in gene flow within the last 500 years, hspAmerind strains are excluded from the donor set because many of the strains have undergone post-Columbian admixture with other populations. The DNA in any case originated from the Old World, albeit probably > 10,000 years ago.

The second global painting panel includes all New World strains, apart from the specific individual being painted. Many, although not all of the chunks inferred to be donated by other New World strains in this painting will represent coalescent events that happened in the New World. Therefore using this painting panel allows us to investigate recent demography within the New World.

**Fig 1:**
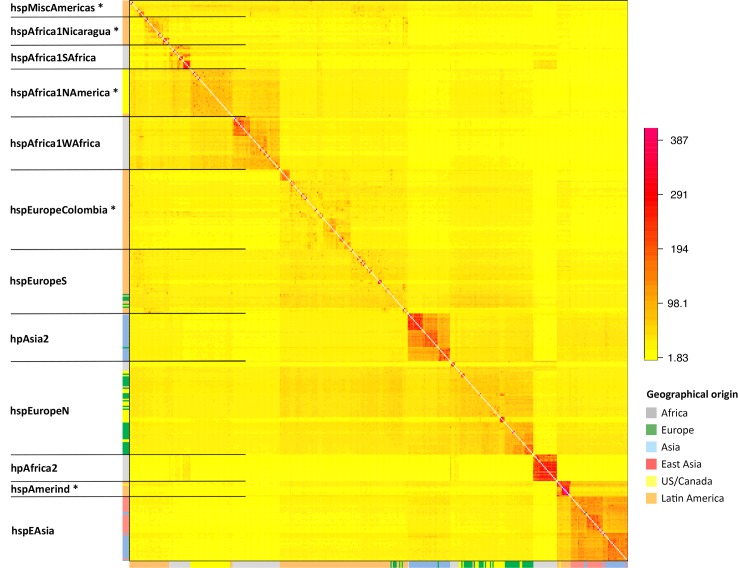
Population structure of global *H*. *pylori* strains. The colour of each cell of the matrix indicates the expected number of DNA chunks imported from a donor genome (column) to a recipient genome (row). The boundaries between named populations are marked with lines, with New World populations marked with an asterisk. The colour bar on the left indicates the geographical locations where the strains were sampled.

**Fig 2:**
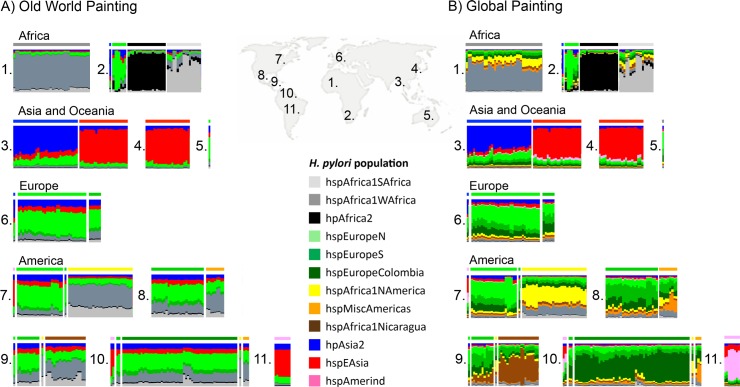
Ancestry of *H*. *pylori* inferred by chromosome painting. Each vertical bar represents one isolate, which are ordered by geographical origin (1–11). 1: West Africa, 2: South Africa, 3: Central Asia, 4: East Asia, 5: Australia, 6: Europe, 7: US and Canada, 8: Mexico, 9: Central America, 10: Colombia, 11: Peruvian Amazon. The colour composition of each bar indicates each of the subpopulations’ contribution to the core genome of that isolate. A) Old world painting where only isolates from Old world areas (1–6 on map) have been used as donors in the chromosome painting. B) Global painting in which all populations have been used as donors.

fineSTRUCTURE uses the output of Chromopainter to assign individuals to populations with distinct ancestry profiles. We applied fineSTRUCTURE to the global painting to identify subpopulations in the dataset (Fig 1). In order to make display and reporting of the results tractable, we merged the most similar populations until 12 distinct populations remained, 5 of which are restricted to the New World. The “palette” of each strain, representing the proportion of chunks that come from each population in the donor panel, was determined for both the Old World (Fig 2A) and the Global (Fig 2B) painting. One of the twelve populations, hspMiscAmerica, contains isolates that are not particularly closely related to each other and should not be thought of as a coherent population (Fig 1). The fineSTRUCTURE results are congruent with those obtained by Principal Component Analysis, PCA, which show differences between the subpopulations within the first 5 Principal Components (S1 Fig) but are easier to interpret.

### Increased number of isolates reveals substructures in the Old World populations

Each of the 7 populations found in the Old World has been reported previously with the exception that, with the addition of the large number of isolates in this study, hpEurope isolates separated into two distinct groups, which we provisionally label hspEuropeN and hspEuropeS (Fig 1). Our geographical sampling within Europe is limited but this split is likely to reflect the previously observed North to South gene frequency cline [12,13], with the hspEuropeS isolates having a larger fraction of their palette from African populations and hspEuropeN having a higher proportion from hpAsia2. The other five populations, hpAfrica2, hspAfrica1SAfrica, hspAfrica1WAfrica, hpEastAsia and hpAsia2 are highly distinct from each other, each receiving more than half of their palette from their own population in the Old World painting.

### Distinct subpopulations of mixed hpEurope and hpAfrica1 ancestry in American *H*. *pylori*

Among the isolates from the Americas, five additional subpopulations could be distinguished; four have palettes consistent with being European/African hybrids, according to the Old World painting (Fig 2A). The population with the highest African ancestry is *hspAfrica1NAmerica*, isolated from 30 individuals in the US, one in Canada, one in Nicaragua, and one in Colombia, followed by *hspAfrica1Nicaragua*, which only contains isolates from Nicaragua; *hspMiscAmerica*, which consists of a number of strains of Mexican and Colombian origin; and *hspEuropeColombia*, which contains most of the Colombian isolates in our data set, and has a palette similar to hspEuropeS (Fig 1). The fifth population, hspAmerind, has a palette similar to hpEastAsia but with more hpEurope ancestry. These results are congruent to those obtained using D statistics (Table 1), which also imply that European and post-Colombian New World subpopulations are hybrids.

**Table 1:** D-statistics.

Population 1	Population 2	Population 3	Population 4	D-statistic
hpAfrica2	hspAfrica1WAfrica	hpAsia2	hspAfrica1NAmerica	0.538
hpAfrica2	hspAfrica1WAfrica	hpAsia2	hspAfrica1Nicaragua	0.456
hpAfrica2	hspAfrica1WAfrica	hpAsia2	hspMiscAmericas	0.454
hpAfrica2	hspAfrica1WAfrica	hpAsia2	hpEuropeColombia	0.289
hpAfrica2	hspAfrica1WAfrica	hpAsia2	hspEuropeS	0.274
hpAfrica2	hspAfrica1WAfrica	hpAsia2	hspEuropeN	0.102
hpAfrica2	hspAfrica1WAfrica	hpAsia2	hspAmerind	-0.058
hpAfrica2	hspAfrica1WAfrica	hpAsia2	hpEastAsia	-0.072

In our sample, several isolates from the Americas cluster within the two hpEurope subpopulations (Fig 1). The hpEurope strains from North America largely cluster with hspEuropeN while those from Central and Southern America cluster with hspEuropeS. There was also substantial variation in the proportion of the genomic palette stemming from hspAfrica1WAfrica and hspAfrica1SAfrica, both between and within New World populations. hspAfrica1WAfrica is the major African source in isolates from hspMiscAmerica, hspEuropeColombia as well as hspEuropeS while hspAfrica1SAfrica is a more important source for hspAfrica1NAmerica and hspAfrica1Nicaragua populations. A handful of isolates from both hspEuropeColombia and hspAfrica1Nicaragua populations have elevated hspAfrica1SAfrica proportions, consistent with recent genetic mixture (Fig 2A).

### The distinct New World subpopulations show evidence of both drift and mixture

**Fig 3:**
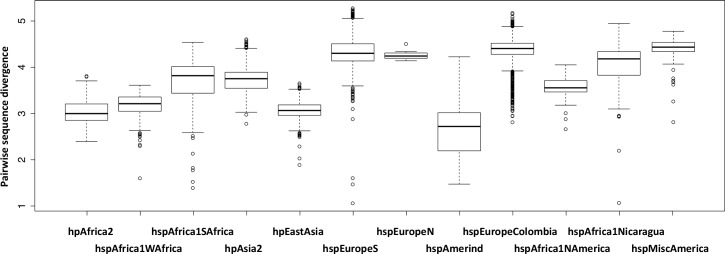
Pairwise sequence divergence within populations. For the two hspEurope populations only the Old World isolates are included.

In the global painting, the strains from the New World populations received a large proportion from their palette from within their own subpopulation, meaning that they have differentiated both from the Old World isolates as well as from the other New World subpopulations. The formation of differentiated populations in the Americas is suggestive of recent demographic bottlenecks (see discussion below) but the New World populations have nucleotide diversity as high as or slightly higher than the Old World populations from which they evolved (Fig 3), presumably because the diversity lost in bottlenecks has been replaced by admixture.

Identifying the components of the ancestry of the New World populations that have undergone higher levels of drift provides insight into the process of differentiation. Drift is likely to be caused by the expansion of particular clones or lineages, for example, due to transmission bottlenecks. Specifically, we focused on signatures of DNA that had the most recent coalescent with other members within the same population. We tabulated the proportion of such sites with each distinct ancestry source in the Old World painting that were inferred to instead be derived from other members of their own population in the New World painting (Table 2). Bottlenecks allow small numbers of clones to propagate, leading to high rates of within population coalescence for genomes sampled from the population. This will in turn increase the proportion of sites inferred to have donors within the same population in the New World painting, rather than from Old World or other sources. Diversity acquired by admixture on the other hand, is more likely to be copied from other populations, unless the admixture sources have themselves been subject to a strong bottleneck.

**Table 2:** Proportion of ancestry assigned to each Old World population (columns) in the Old World painting that have a more recent common ancestor within the same subpopulation in the Global Painting.

	hspAfrica1-SAfrica	hspAfrica1-WAfrica	hspEuropeS	hspEuropeN	hpAsia2	hpEastAsia
hspEuropeColombia	0.43	0.44	0.48	0.48	0.46	0.39
hspAfrica1NAmerica	0.42	0.41	0.34	0.27	0.27	0.29
hspAfrica1Nicaragua	0.61	0.66	0.43	0.45	0.47	0.50
hspMiscAmerica	0.11	0.21	0.06	0.06	0.06	0.06
hspAmerind	0.46	0.45	0.44	0.47	0.50	0.53

For hspAfrica1NAmerica and hspAfrica1Nicaragua, the most drifted component is the African component. The level of drift of the African component is significantly higher than that of other components (p < 10^−15^ and p < 10^−8^ by Wilcoxon’s rank sum test in hspAfrica1NAmerica and hspAfrica1Nicaragua, respectively). It suggests that African lineages may have undergone rapid demographic increases during their spread in the Americas and thus that they may have a transmission advantage.

Aside from the isolation of hspAmerind strains from three countries and a single hspAfrica1NAmerica isolate from Colombian and Nicaraguan, there was no indication of sharing of ancestry between North, Central and South American gene pools. There is also no evidence from the palettes of hspAmerind having contributed DNA to any other New World strains. Amongst the Mexican isolates, a few hspMiscAmerica isolates have a substantial hspAfrica1NAmerica component but there is no sign of elevated ancestry from the Colombian or Nicaraguan populations.

The palettes provide evidence of genetic mixture between populations within countries. The hspEuropeS isolates from Nicaragua have more hspAfrica1Nicaragua in their palette than those from other locations, while Colombian isolates that are not assigned to the hspEuropeColombia have a higher ratio of hspEuropeColombia/hspEuropeS than found elsewhere, which is consistent with recent genetic exchange. Conversely, there is no evidence for elevated hspAfrica1NAmerica ancestry in hspEuropeN isolates from North America. The hspAfrica1NAmerica population has more hpEurope ancestry than hpAfrica1 isolates from Africa but there is little variation between strains, contrary to what would be expected if there was substantial ongoing gene flow.

### Several genes have ancestral origin distinct from the overall core ancestry

The spread of *H*. *pylori* populations in the Americas provides an opportunity to investigate adaptive introgression as the bacteria encountered new populations of humans, as well as novel diets and environmental conditions. This is of specific interest since *H*. *pylori* has an outstanding capacity for recombination between co-colonising strains [2,14]. We performed a scan of the core genome for genomic regions with enrichment of specific ancestry components. To this end, we painted the strains from each New World population, using Old World strains as donors and recorded whether the donor was European, African or Asian in origin.

We found several genes where alleles showed significantly higher or lower ancestry from another Old world donor population than would be expected based on the overall ancestry of that isolate (p < 10^−8^, Table 3). Among these were three genes that had ancestry from an unexpected Old World source in more than one of the New World populations. These were the genes encoding for AlpB (HP0913), HofC (HP0486), and FrpB4 (HP1512), which notably all are outer membrane proteins (S1 Fig) and all enriched for Asian ancestry in at least one population. The regions in *alpB* (S1A Fig) consist of clusters of 24 and 32 polymorphic sites enriched for Asian ancestry (lowest p-value 9.8 x 10^−15^) within 49 and 65bp in hspEuropeColombia and hspAfrica1Nicaragua populations, respectively. The regions in *hofC* (S1B Fig) consist of 2 SNPs with interval 171bp and 4 successive SNPs enriched for Asian ancestry (lowest p-value 7.8 x 10^−15^) in hspEuropeColombia and hspAfrica1NAmerica populations, respectively. The regions in *frpB4* (S1C Fig) consist of 2 successive SNPs and 26 SNPs within 156 bp enriched for Asian ancestry (lowest p-value 3.5 x 10^−10^) in hspEuropeColombia and hspAfrica1Nicaragua populations, respectively.

**Table 3:** Genes carrying position(s) with enrichment of a specific ancestry components.

Locus tag	Gene	Description	Population showing the enrichment	Enrichment	P-value*
HP0026	*gltA*	type II citrate synthase	hpEuropeColombia	Asia_high	1.4E-10
HP0042	* *	trbI protein	hpEuropeColombia	Africa_high	1.0E-09
HP0099	*tlpA*	methyl-accepting chemotaxis protein	hspAfrica1NAmerica	Africa_low	1.2E-09
HP0160	* *	hypothetical protein	hpEuropeColombia	Europe_high	4.1E-12
HP0216	* *	1-deoxy-D-xylulose 5-phosphate reductoisomerase	hpEuropeColombia	Africa_high	3.9E-10
HP0252	* *	hypothetical protein	hpEuropeColombia	Africa_low	4.3E-09
	* *			Europe_high	2.8E-13
HP0272	* *	hypothetical protein	hpEuropeColombia	Europe_high	3.4E-10
HP0408	* *	hypothetical protein	hpEuropeColombia	Europe_high	2.1E-13
HP0486	*hofC*	outer membrane protein	hpEuropeColombia	Asia_high	7.3E-09
	* *			Europe_high	1.2E-10
	* *		hspAfrica1NAmerica	Asia_high	7.8E-15
	* *		hspAfrica1Nicaragua	Europe_high	8.8E-11
HP0492	* *	hypothetical protein	hpEuropeColombia	Europe_high	9.0E-09
HP0568	* *	hypothetical protein	hpEuropeColombia	Africa_high	2.2E-09
HP0597	* *	penicillin-binding protein 1A (PBP-1A)	hpEuropeColombia	Africa_high	9.5E-10
HP0605	* *	hypothetical protein	hpEuropeColombia	Asia_high	8.8E-11
HP0607	*hefC*	acriflavine resistance protein	hpEuropeColombia	Africa_low	2.4E-09
HP0610	* *	toxin-like outer membrane protein (vacA paralog)	hpEuropeColombia	Europe_high	3.1E-09
HP0667	* *	hypothetical protein	hpEuropeColombia	Africa_high	3.5E-09
HP0872	*phnA*	alkylphosphonate uptake protein	hpEuropeColombia	Asia_high	3.9E-11
HP0913	*alpB/hopB*	outer membrane protein Omp21	hpEuropeColombia	Asia_high	9.8E-15
	* *		hspAfrica1Nicaragua	Asia_high	1.1E-12
HP0953	* *	hypothetical protein	hpEuropeColombia	Europe_high	2.5E-11
HP0978	* *	cell division protein (ftsA) protein	hpEuropeColombia	Africa_low	4.6E-09
HP1055	* *	hypothetical protein	hpEuropeColombia	Africa_high	4.8E-09
HP1086	* *	hemolysin (tly)	hpEuropeColombia	Europe_high	2.8E-10
HP1156	* *	hypothetical protein	hspAfrica1NAmerica	Africa_low	6.8E-09
HP1339	*exbB*	biopolymer transport protein	hpEuropeColombia	Africa_low	5.5E-09
	* *		hpEuropeColombia	Europe_high	8.2E-15
	* *		hspAfrica1Nicaragua	Europe_high	1.4E-12
HP1395	* *	hypothetical protein	hpEuropeColombia	Europe_high	6.9E-10
HP1487	* *	hypothetical protein	hpEuropeColombia	Africa_high	5.5E-09
HP1512	*frpB4*	iron-regulated outer membrane protein	hpEuropeColombia	Asia_high	3.6E-10
	* *		hspAfrica1Nicaragua	Asia_high	3.5E-10
	* *		hspAfrica1Nicaragua	Europe_high	4.1E-13

*the lowest P-value among polymorphic sites in a gene

**Fig 4:**
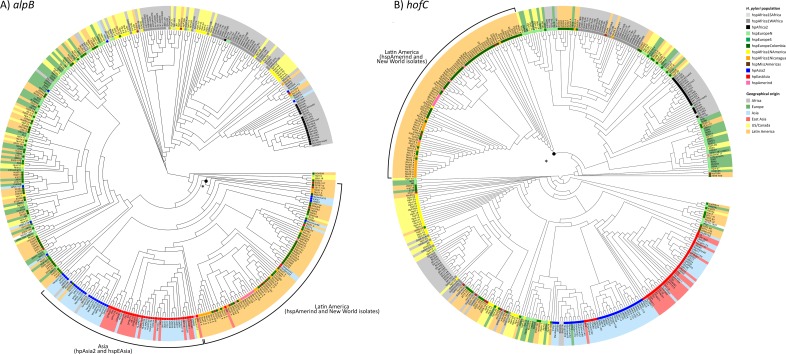
Maximum likelihood phylogenetic trees of alpB and hofC genes. Leaves are shaded according to geographical origin and the *H*. *pylori* population assignment according to the FineSTRUCTURE analysis is marked at the base of each leaf. A) AlpB. A black dot with an asterisk marks the branch for which the joint Latin American and Asian clade segregate from the others. B) HofC. The black dot with an asterisk marks the branch at which the South American clade segregates from the others.

To investigate the basis of the low p values in more detail, we first constructed phylogenetic trees of the three genes. Linkage disequilibrium extends over very short distances in *H*. *pylori* so these trees do not necessarily reflect the genealogy of the gene as a whole. Nevertheless interesting patterns were found in *alpB* and *hofC* trees (Fig 4). For each gene at least one major separate clade of Latin American isolates could be observed, regardless of *H*. *pylori* population. The tree for *frpB4* can be found in S2 Fig.

For *alpB* there are three major clusters; one predominantly Asian cluster including a majority of the Latin American strains, both Amerind isolates and isolates from the New World subpopulations, one predominantly European cluster, also with a number of Latin American strains, and one African cluster where isolates from Africa group together with isolates the hspAfrica1NAmerica. Notably, in the Asian group the Latin American isolates from multiple *H*. *pylori* populations cluster together while in the European group they are interspersed with the other isolates (Fig 4A).

For *hofC* there is one clearly distinct South American clade, including all the Amerindian strains except for Aklavik117 and a majority of the strains belonging to the New World subpopulations hspMiscAmericas, hspAfrica1Nicaragua and hspEuropeColombia. The other three main clades are dominated by either: (*i*) hspAfrica1WAfrica, hpAfrica2 and hspAfrica1NAmerica isolates; (*ii*) hspAfrica1SAfrica, European and US/Canadian hpEurope isolates or; (*iii*) Asian isolates, respectively (Fig 4B). Notably, for *hofC* the Mexican isolates did not group within the main South American clade but within clade *i* and *ii*.

Investigating the *hofC* gene alignment in more detail using F_st_ values revealed that the sequence variation strongly contributing to the tree clade structure were nucleotides 826–926 of the gene. We found 10 nucleotide positions with a Fixation index of higher than 0.3 in the Latin American isolates compared to isolates from rest of the World (S4A Fig), out of which the 8 highest were localized in the above-mentioned region. Notably, these F_st_ values were also among the highest out of all nucleotide positions in the core genome (Table 4).

**Table 4:** The ten core genome positions of highest Fst values in Latin American isolates compared to the rest of the World.

Locus tag	Gene	Description	Position in 26695	Fst
HP0486	hofC	Outer membrane protein HofC	879	0.61
HP1339	exbB	Biopolymer transport protein ExbB	112	0.61
HP0486	hofC	Outer membrane protein HofC	885	0.61
HP0486	hofC	Outer membrane protein HofC	971	0.60
HP0486	hofC	Outer membrane protein HofC	972	0.60
HP0486	hofC	Outer membrane protein HofC	967	0.59
HP0486	hofC	Outer membrane protein HofC	970	0.59
HP0486	hofC	Outer membrane protein HofC	921	0.56
HP0175	ppiC	Putative peptidyl-prolyl cis-trans isomerase PpiC	550	0.44
HP0175	ppiC	Putative peptidyl-prolyl cis-trans isomerase PpiC	636	0.44

**Fig 5:**
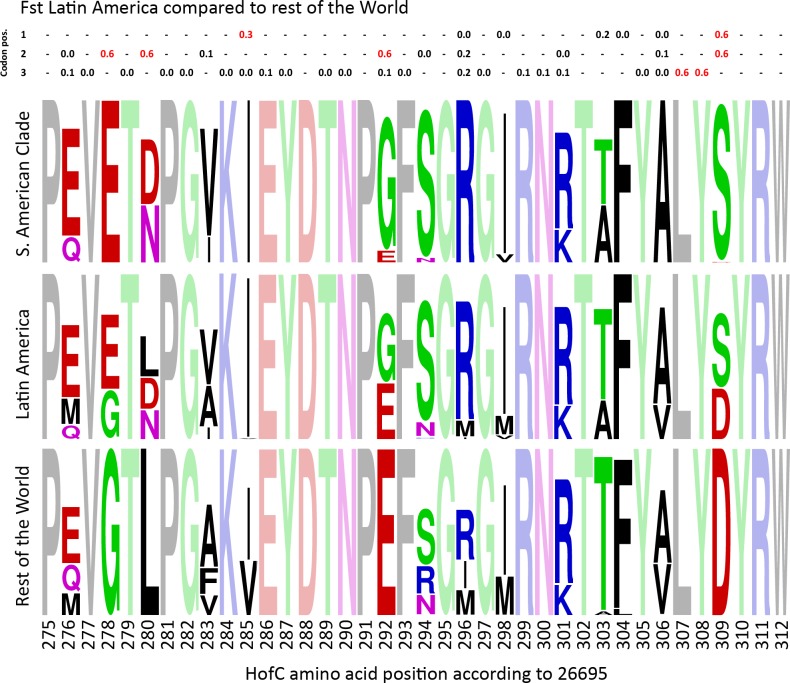
Fst values and WebLogo representations for amino acids 275–312 of HofC. The upper rows are Fst values for the triplets of nucleotides in each codon, calculated for isolates originating from Latin America compared to isolates from the rest of the world. WebLogo representations of the region are showed for i) the South American clade in Fig 4B, ii) All isolates from Latin America (for which the Fst was calculated), and iii) Isolates from the rest of the world. Shaded residues are synonymous in all three populations.

Within this stretch, several amino acids were completely fixed in the South American clade and were not found in the other isolates (Fig 5). The ones with strongest F_st_ and unique to the South American clade were a Glutamic acid instead of a Glycine at position 278, Asparagine or Aspartic Acid instead of Leucine at position 280, a strong tendency to have Glycine instead of Glutamic Acid at position 292 and a Serine instead of Aspartic Acid at position 309 (Fig 5). These changes, which in most of the cases entirely changes the residue characteristics have spread to a large proportion of isolates in all of the populations found in South America, suggesting they confer an adaptive advantage, and stand out strongly in the F_st_ analyses even though this includes all Latin American isolates and not only the specific clade in the tree.

### Accessory genome analysis shows similar but not identical ancestral patterns to the core genome

Our collection of multiple genomes from each population allowed us to examine patterns of gene presence and absence. A neighbour-joining tree based on gene sharing distance between isolates largely recovered the populations and sub-populations identified based on core genome sequence, but with distinct clusters for isolates carrying the Cag Pathogenicity Island (cagPAI) positive and for cagPAI negative isolates respectively (S5 Fig). The *cagPAI* is an approximately 40 kb cluster of genes encoding for a Type IV secretion system. This secretion system is translocating the CagA protein into host cells and has been shown to be of high importance for bacterial virulence [15,16].

**Fig 6:**
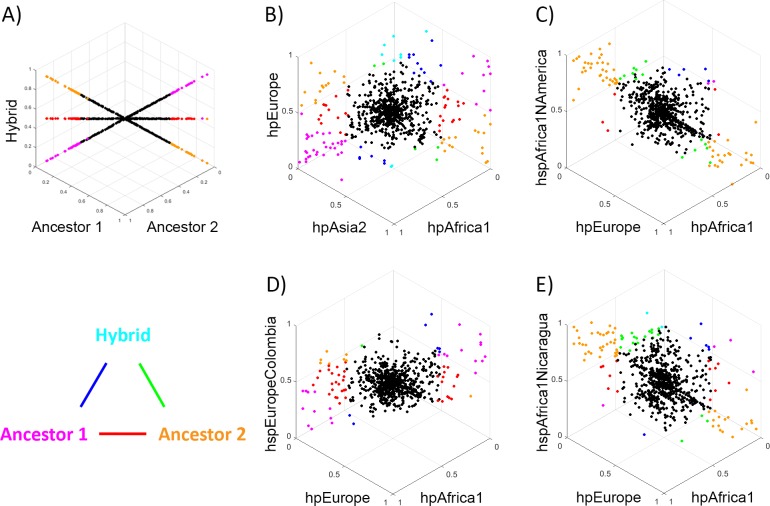
Comparison of accessory gene frequency in a hybrid population with the frequencies in its putative ancestors. Each dot shows the frequencies of an accessory gene in three populations, with the graphs orientated such that genes with identical frequencies in all three appear at the centre of the plot. Genes with large frequency differences between populations are labelled in colours, according to the triangular legend. Colours shown on the vertices indicate genes that differ substantially between one population and the other two (according to the criteria that X is considered substantially bigger than Y if X–Y > = 0.5, X > = 0.5 and Y < 0.1, or X > 0.9 and Y < = 0.5), while colours on the edges indicate genes where the two populations on the vertices differ substantially in frequency, with the third population having an intermediate frequency. A) Plot showing results obtained if the frequency of genes in the hybrid population is either identical to Ancestor 1 (line ending in magenta), to Ancestor 2 (line ending in orange) or a 50–50 hybrid (line ending in red). B) Comparison between Old world populations hpEurope, hpAsia2 and hpAfrica1, C) Comparison of hspEuropeColombia to hpEurope and hpAfrica, D) Comparison of hspAfrica1Nicaragua to hpEurope and hpAfrica, E) Comparison of hspAfrica1NAmerica to hpEurope and hpAfrica.

In order to assess whether the pan genome evolved by the same processes of clonal descent and genetic exchange as the core genome, we examined the frequency of different pan genome elements in different populations. Specifically, we jointly analysed the frequency genes of triplets of populations, two of which are close representatives of the presumed ancestral source population and a third putative hybrid, with projections of the resulting 3D plots shown in Fig 6. Fig 6A shows the expectations if the pan genome of the descendent population had identical gene frequencies to either source or a 50–50 hybrid.

It has been previously shown that for the core genome, hpEurope bacteria are hybrids between hpAsia2 and hpNEAfrica (which is related to hpAfrica1), with higher hpAsia2 ancestry proportions in Northern Europe [12,17]. The same pattern for the pan genome could also be observed in our analysis, where the hpEurope population has a profile that is intermediate between that of hpAsia2 and hpAfrica1, but with considerable variation in the pattern amongst genes, consistent with genetic drift in the thousands of years since hybridization (Fig 6B, S1 Movie). We confirmed this visual impression using an ANOVA (S3 Table). Specifically, we tabulated the genes that differed in frequency amongst the three populations and found that the average deviations from equality were largest for genes with pattern showing either hpEurope being similar in frequency to hpAsia2 or hpEurope being similar in frequency to hpAfrica1.

For the New World populations, hspEuropeColombia has a profile that is intermediate between Africa1 and European isolates (Fig 6D, S3 Movie), with the ANOVA implying that gene frequencies are more similar to hpEurope than to hpAfrica1 (S3 Table). hspAfrica1Nicaragua and hspAfrica1NAmerica have pan genomes that are more similar to those of hpAfrica1 than hpEurope (Fig 6C–6E, S2 and S4 Movies, S3 Table).

## Discussion

Millions of people from diverse geographical and ethnic backgrounds have migrated from the Old World to the Americas in the last 500 years and it is likely that a majority carried *H*. *pylori*. Transmission between ethnicities and DNA exchange between strains might be expected to scramble the relationship between bacterial and human ancestry at the individual level, but in the absence of selection or bottlenecks, overall *H*. *pylori* ancestry should largely recapitulate the ancestry found in humans [12,17,18]. Consistent with this expectation, we find diverse populations of hpEurope bacteria in Northern and Latin America, with chromosome painting profiles comparable to those found in European isolates. We find a broad North-South divide amongst hpEurope isolates, both in the New and Old World, with higher relatedness to hpAfrica1 DNA in the southern populations. This is consistent with the gene frequency cline already observed in Europe and known differences in the colonization history of North and South America [19].

However, *H*. *pylori* genomic variation does not necessarily recapitulate patterns found in humans. The Americas constituted both a new physical and dietary environment and a new ethnic mix of hosts. Particular bacterial lineages may have had, or acquired, traits that adapted them to these new conditions. In extreme cases, human migrations that have little or no effect on human ancestry might precipitate substantial changes in *H*. *pylori* populations. For example, hspAmerind strains are rare even in populations with substantial Native American ancestry [3]. This suggests that after more than 10,000 years of independent evolution, resident *H*. *pylori* lineages were poorly equipped to compete with incoming lineages or with changes in the environment caused by the new settlers. We also found evidence of substantial differentiation of New World *H*. *pylori* populations from their ancestors, which suggests that there have been bottlenecks with particular lineages contributing disproportionately to extant populations. These bottlenecks have most strongly affected African components of ancestry (Table 2), suggesting that bacteria of African origin may have been particularly effective in colonizing the new continent.

We identified three differentiated populations in the Americas, in addition to hspAmerind. The hspAfrica1NAmerica population includes the non-European isolates from the US, also found in single Canadian, Colombian and Nicaraguan isolates. This population has an ancestry profile consistent with it being a mix of West African, South African and European sources. However, our global chromosome painting results (Fig 2B) show that within genomic regions of African origin, the DNA of hspAfrica1NAmerica is distinct from that found in modern Gambian and South African populations. Differentiation at the DNA sequence level is also found in the hspEuropeColombia and hspAfrica1Nicaragua populations, whose gene pools are distinct from each other and from those in Mexico and Europe.

The three larger groups of samples, from Mexico (Mexico City), Nicaragua (Managua) and Colombia (Bogotá) respectively, were all collected at hospitals that are tertiary referral centres for endoscopy with large catchment areas, while all but one of the US isolates came from a hospital in Cleveland, a cosmopolitan city. Therefore, our findings likely reflect broad patterns of diversity within large geographic regions. Within our sample, there are regional differences in the proportions of European, African and Amerind ancestry and wider sampling might have differentiated the picture further. Nevertheless, the distinct patterns of *H*. *pylori* ancestry in the four countries indicate that recent population movements have been strongly influenced by national boundaries.

*H*. *pylori* can undergo high levels of recombination during mixed infection. Over time, this might lead to bacteria acquiring an ancestry profile that reflects their local gene pool rather than their continent of origin. Recombination has not proceeded this far anywhere in the Americas and multiple populations with distinct ancestry profiles are found in most locations. hspAmerind strains have not contributed substantially to the ancestry of bacteria from any other population, but do appear to have acquired hpEurope DNA themselves. In Nicaragua and Colombia, recombination has transmitted distinctive DNA between populations, e.g. the brown shaded component in the hspEuropeS isolates from Nicaragua (Fig 2B), leading to what can informally be thought of as a national signature in the *H*. *pylori* DNA. There is no equivalent signal of hspAfrica1NAmerica DNA amongst the hpEurope bacteria from the US, indicating that recombination between these populations has been less extensive, and there is also no evidence within our sample of a distinctive population of hpEurope bacteria evolving within the US. Similar patterns of higher admixture in African American and Hispanic American individuals than in American individuals of European descent have been observed also on human genomic level [19].

The differences in the extent of admixture in the New World populations can have several explanations including differences in dates of colonization and extent of European and African influx/admixture in Latin America compared to the US. Another important factor can be the prevalence of infection in different areas. The prevalence of *H*. *pylori* infection remains high in Latin American countries, ranging from 70.1% to 84.7% of adults in a recent multi-country study [20]. In the US, the prevalence has been declining from high levels and according to data from the end of the 1990’s, is around 32.5% [21]. The prevalence was different between the ethnic groups: 52.7% in non-Hispanic blacks; 61.6% in Mexican Americans and; 26.2% in non-Hispanic whites [21]. High prevalence likely entails higher occurrence of horizontal transmission and mixed infections and thus the possibility of recombination between distantly related strains [22] [23].

Our sample of Old World sources is incomplete, both in Africa and Europe, and therefore it is likely that Old World sub-populations exist that are more closely related to the New World populations than those in our sample, one such area being the Iberian peninsula. Also, even if we sample extensively in modern human groups, this may not fully reflect structure 500 years ago. The absence of sampling of close surrogates of the true ancestral subpopulations may alter our conclusions about selection or drift, which we have interpreted to have taken place in the New World rather than in the Old World. Sampling limitations for example make it unclear how much of the extensive mixture between African and European DNA observed in many Central and Southern American isolates actually took place in the Americas. Nevertheless, it is difficult to explain the local affinities within the diverse gene pools in both Nicaragua and Colombia, except by local genetic exchange. The hspAfrica1NAmerica isolates are homogeneous in their ancestry profile, suggesting that they also form a distinct gene pool that has acquired its characteristics through substantial evolution within the USA, although some of this evolution may have happened in an as yet unsampled subpopulation in Africa.

hspAfrica1NAmerica appears to be an approximately panmictic population. For example, all isolates have approximately the same level of hpEurope ancestry in Fig 1. This feature is difficult to reconcile with the low levels of genetic exchange observed with hpEurope isolates from the US. Since it has been shown that *H*. *pylori* from the same population (hpEastAsia) can exchange 10% of their genome during a single four year mixed infection in human [24], the ancestral pattern in US *H*. *pylori* implies barriers to recombination between the two populations. Such barriers may be the result of ethnic segregation and thus less diverse co-infections, of differential uptake or incorporation of DNA from different populations, or of efficient competitive exclusion of bacteria from one population by bacteria from the other within individual stomachs.

In the New World populations, four genes encoding for outer membrane proteins have sequence with ancestry that differed from that inferred for the overall core genome in more than one of the New World population. Interestingly, several of these variants were common for Latin American isolates regardless of which ancestral population they belonged to. AlpB is an adhesin binding to laminins in the extracellular matrix [25] that is present in all *H*. *pylori* strains [26]. Together with AlpA, it is required for colonization in experimental models and for efficient adhesion to gastric epithelial cells [27]. The HofC protein is also required for *H*. *pylori* colonization in mice and gerbils [28,29] but is not well characterized and little is known about its function. FrpB4 is important in the bacterial adaptation to variation in the microenvironment. FrpB4 is regulated by the levels of nickel, a micronutrient essential for *H*. *pylori* survival, growth and expression of virulence factors in the human stomach [30–32].

The enrichment pattern in *hofC* in a high number of the South American isolates was largely explained by the positions in region 276–309 of the 528 amino acid protein. The variants were found in all the South American Amerindian strains as well as almost all of the hspAfrica1Nicaragua and a majority of hspEuropeColombia strains together with strains from Peru and El Salvador. No Mexican strains were found in this clade. Since the HofC protein structure and function are not characterised in detail, we are unfortunately unable to predict how these alleles contribute to the function or specificity of the protein. Interestingly, also in FrpB4 there were several positions of high Fst in Latin America compared to the rest of the world (S4 Fig) but nor in this case we are able speculate in the functional impact of these specific positions. Nevertheless, the very pronounced enrichment pattern, as well as that in the other genes, is consistent with the New World *H*. *pylori* having adapted to their respective human populations, allowing certain traits to propagate relative to the overall genetic background. This could be important in understanding the differences in pathogenicity in different areas and different host/bacterial interactions, suggesting a need for further investigation of the function of these proteins.

Our analysis of the accessory genome shows that *H*. *pylori* gene content, as well as nucleotide composition, is mixed during admixture between host populations. For example, the gene content of hpEurope is intermediate between that of hpAfrica1 and hpAsia2, but with substantial variation that may reflect the large time that has elapsed since admixture. hspEuropeColombia is more African in genome content than the average hpEurope bacteria from Europe, as would be expected because of its higher African ancestry at the nucleotide level. However, the genome content of strains from the hspAfrica1Nicaragua population is more African than would be expected given its substantial co-ancestry with hpEurope within the core genome. This observation is concordant with recent observations showing that restriction modification inhibits non homologous but not homologous recombination [33], suggesting that core genome ancestry may mix more readily between populations than accessory elements if restriction modification is an important barrier to exchange.

Our results on the population structure in the Americas sheds new light on the relationship between human migration and *H*. *pylori* diversity. In particular, we show that at least during human population upheavals, evolution within geographic locations is far more dynamic than the broad correlation with human genetic variation would suggest and that novel subpopulations can arise by a combination of genetic drift and admixture within hundreds of years.

## Materials and methods

### *Helicobacter pylori* whole genome sequencing data

We used both publicly available and newly sequenced genomes of *H*. *pylori* isolates, 401 in total (S1 Table). Nicaraguan isolates were collected at Hospital Escuela Antonio Lenin Fonseca (HEALF) in Managua, within the international collaboration “Immunological Biomarkers in Gastric Cancer development” and previously described in [34]. Colombian isolates that are not previously described were collected at the Oncology hospital (INCAN) in Bogota, and the Mexican isolates were collected at the Oncology and General Hospital in Mexico City. All three hospitals are tertiary referral centres for endoscopy and patients may thus come from other locations within the countries. For the cases we had more detailed data on the origin of the individuals, this is noted in S1 Table.

The publicly available Colombian and North American genomes were those reported in preceding studies, i.e [35–37].

### Data preparation

All of the genome sequences were imported into the Bacterial Isolate Genome sequence database (BIGSdb) [38]. After this, a gene-by-gene alignment was performed using CDS sequences of the *H*. *pylori* 26695 strains as reference, and the alignments were exported from the database. Both the genome sequences and the alignment are available at the public data repository Dryad (http://datadryad.org/), with doi doi:10.5061/dryad.8qp4n. We conducted SNP calling for each alignment, and imputation for polymorphic sites with missing frequency < 1% using BEAGLE [39] as our preceding study [40]. We combined in total 401350 SNPs in 1232 genes while preserving information of SNP positions in the reference genome, to prepare genome-wide haplotype data.

### Population structure analysis

We inferred population structure among the strains from the genome-wide haplotype data by using the chromosome painting and fineSTRUCTURE [9], according to a procedure of our preceding study that applied them to *H*. *pylori* genomes [10]. Briefly, we used ChromoPainter (version 0.04) to infer chunks of DNA donated from a donor to a recipient for each recipient haplotype, and summarized the results into a “co-ancestry matrix” which contains the number of recombination-derived chunks from each donor to each recipient individual. We then ran fineSTRUCTURE (version 0.02) for 100,000 iterations of both the burn-in and Markov chain Monte Carlo (MCMC) chain in order to conduct clustering of individuals based on the co-ancestry matrix.

Principal Component Analysis was performed by applying the standard PCA implemented in Eigensoft to our data (more precisely, all biallelic data after pruning of SNPs with r2 > 0.7).

D-statistics were calculated by using popstats (https://github.com/pontussk/popstats) and specifying POP1 as hpAfrica2, POP2 as hspAfrica1WAfrica, POP3 as hpAsia2, and POP4 as either of the remaining 9 populations, respectively.

### Stratified chromosome painting

We conducted two types of chromosome painting; “Old World chromosome painting” using only Old world isolates as donors, and “Global chromosome painting” in which each isolate is painted using all of the others. For this purpose, we used ChromoPainterV2 software [9].

To identify genomic regions with enrichment of unexpected ancestry components in the New World populations hspAfrica1NAmerica, hspAfrica1Nicaragua, and hspEuropeColombia, we conducted a novel statistical test for each of the 401350 SNPs. This was done using the Old world strains as donors, grouped into African, Asian and European geographic origin respectively.

We aim to count the number of recipient haplotypes from a certain donor population at each SNP. However, we do not observe whether a recipient *i* uses a particular donor population a, but instead the probability that it does at each locus *l*. The distribution of the total number of isolates at locus *l* from donor population *a* is ~ Poisson-Binomial(*p*_*lia*_). If we let the genome-wide painting probability be $p_{ia}=(\sum_{l=1}^{L}p_{lia})/L$, then the distribution expected under the null that there is no local structure to the painting donors is ~ Poisson-Binomial(*p*_*ia*_). We therefore report the p-values to test whether locus *i* has significantly enriched for donor *a* (and likewise to test for de-enrichment). We used P<10^−8^ as a significance level, which corresponds to P<0.05 after Bonferroni correction.

Because a) the variance of a Poisson-Binomial is highest when is close to 0.5, and b) the distribution is discrete, this statistic has less power to detect high ancestry contributions from components that have high genome-wide ancestry, especially when sample size is small. In practice this has limited our power to detect regions that have an excess of African ancestry.

### Phylogenetic analysis of genes with enriched ancestry

Multiple alignments of the genes were performed using MUSCLE [41] and the alignment manually inspected to remove sequences with incomplete coverage before a PhyML maximum likelihood tree was created using the SeaView software [42]. All trees were visualized using Evolview [43].

### Fixation index (Fst) analysis

Fixation index (Fst) analysis was performed using the R package PopGenome [44]. For all the 1232 core-genome multiple alignments were converted to VCF format using SNP-sites [45] and site-wise Fst was calculated over all biallelic sites for the subpopulation consisting of all isolates that were geographically originating from Latin America. In total 164 358 positions in 933 of the genes were eligible for the analysis. Of those 187 positions had an Fst of more than 0.25 in the Latin American isolates compared to strains from the rest of the World (S2 Table). WebLogo plots were generated using [46].

### Analysis of gene presence/absence and accessory genome

A pan-genome was constructed with all loci present in at least one of our 401 strains to examine presence/absence of all *H*. *pylori* genes. This pan-genome list of 2462 genes was used as queries of BLASTN against each genome analysed in this study through the BIGSdb Genome Comparator pipeline [38]. Gene presence was judged by a BLASTN match of ≥70% identity over ≥50% of the locus length [47].

### Accessory presence/absence tree

The Genome Comparator Output matrix obtained with BIGSdb was used to build a distance matrix (MATLAB R2015a, The MathWorks, Inc., Natick, Massachusetts, United States). A tree was obtained using SplitsTree4 [48] and was visualised with Evolview [43].

## Supporting information

S1 Fig PCA plots describing the relationships between populations. (TIF)

S2 Fig P-values for enrichment of European and Asian ancestry over genes. Each dot corresponds to a polymorphic site that was tested statistically. The three genes in Table 3 satisfying significance level p < 10^−8^ (p < 0.05 after Bonferroni correction) in more than one of the New World populations are shown. Blue symbols indicate the strength of statistical evidence for Asian enrichment and green European enrichment. Gaps represent sites where the missing frequency > 1% and sites in non-coding regions. A) alpB, B) hofC, and C) frpB4.
(TIF)

S3 Fig Maximum likelihood phylogenetic trees of the frpB4 gene. Leaves are shaded according to geographical origin and the *H*. *pylori* population assignment to according to the FineSTRUCTURE analysis is marked at the base of each leaf.
(TIF)

S4 Fig Fst over the hofC and frpB4 genes. Each dot represents a nucleotide position. For positions with Fst > 0.25 the nucleotide position in 26695 is denoted.
(TIF)

S5 Fig Accessory genome tree. Neighbour-joining tree based on gene sharing distance (absence and presence of genes). The outer circle shows the Old World chromosome painting as in Fig 2A. Circles denote geographical origin and squares the *H*. *pylori* population assignment according to the FineSTRUCTURE analysis. Red stars are marking strains without the Cag Pathogenicity Island (CagPAI)
(TIF)

S1 Table Detailed information of isolates included in the study. (XLSX)

S2 Table Fst values of over 0.25 in comparison of Latin American isolates with those from the rest of the World. (XLSX)

S3 Table Comparisons between scenarios in Fig 5B, based on hpEurope as a hybrid between hpAsia2 and hpAfrica1. (XLSX)

S1 Movie Comparison between Old world populations hpEurope, hpAsia2 and hpAfrica1. (AVI)

S2 Movie Comparison of hspEuropeColombia to hpEurope and hpAfrica. (AVI)

S3 Movie Comparison of hspAfrica1Nicaragua to hpEurope and hpAfrica. (AVI)

S4 Movie Comparison of hspAfrica1NAmerica to hpEurope and hpAfrica. (AVI)
