# Correction: Rapid evolution of distinct *Helicobacter pylori* subpopulations in the Americas

**DOI:** 10.1371/journal.pgen.1006730

**Published:** 2017-04-14

**Authors:** Kaisa Thorell, Koji Yahara, Elvire Berthenet, Daniel J. Lawson, Jane Mikhail, Ikuko Kato, Alfonso Mendez, Cosmeri Rizzato, María Mercedes Bravo, Rumiko Suzuki, Yoshio Yamaoka, Javier Torres, Samuel K. Sheppard, Daniel Falush

The following information is missing from the Funding section: This work was supported by the Grants-in-Aid for Scientific Research from the MEXT (Ministry of Education, Science, Sports and Culture) (15K21554 to KY), a Wellcome Trust Career Development fellowship and funding under the MRC-CLIMB initiative (to SKS and DF), and a United States National Institutes of Health Research grant R21CA182822 (to IK). DJL is funded by Wellcome Trust and Royal Society grant WT104125AIA. The funders had no role in study design, data collection and analysis, decision to publish, or preparation of the manuscript.
