# Supplementary figures and images for: Rapid evolution of distinct Helicobacter pylori subpopulations in the Americas
Source: PLoS Genet. 2017 Feb 23;13(2):e1006546. doi: 10.1371/journal.pgen.1006546 (PMC5322909; doi:10.1371/journal.pgen.1006546)

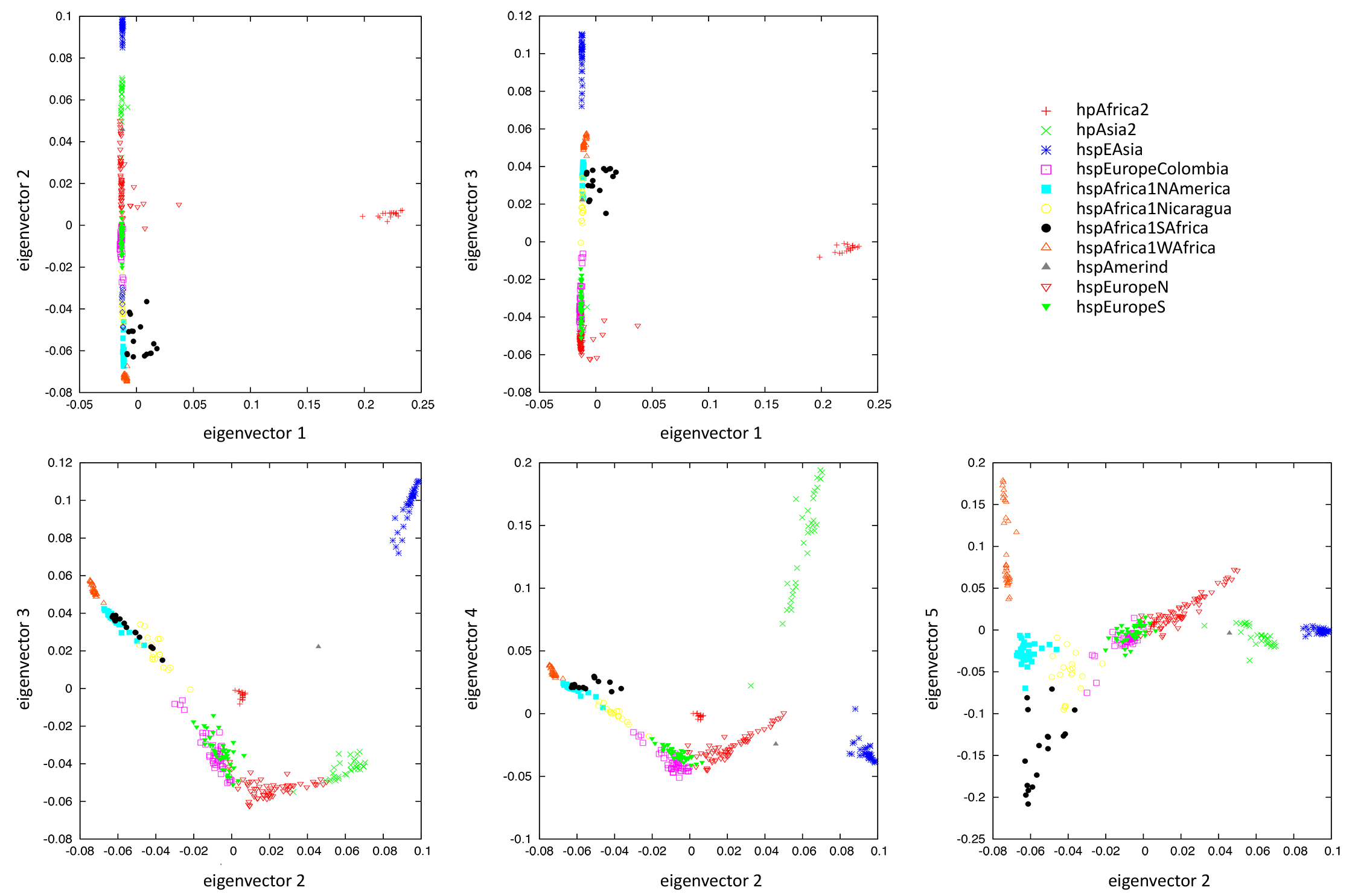

Supplement: S1 Fig — (TIF) [file pgen.1006546.s001.tif]

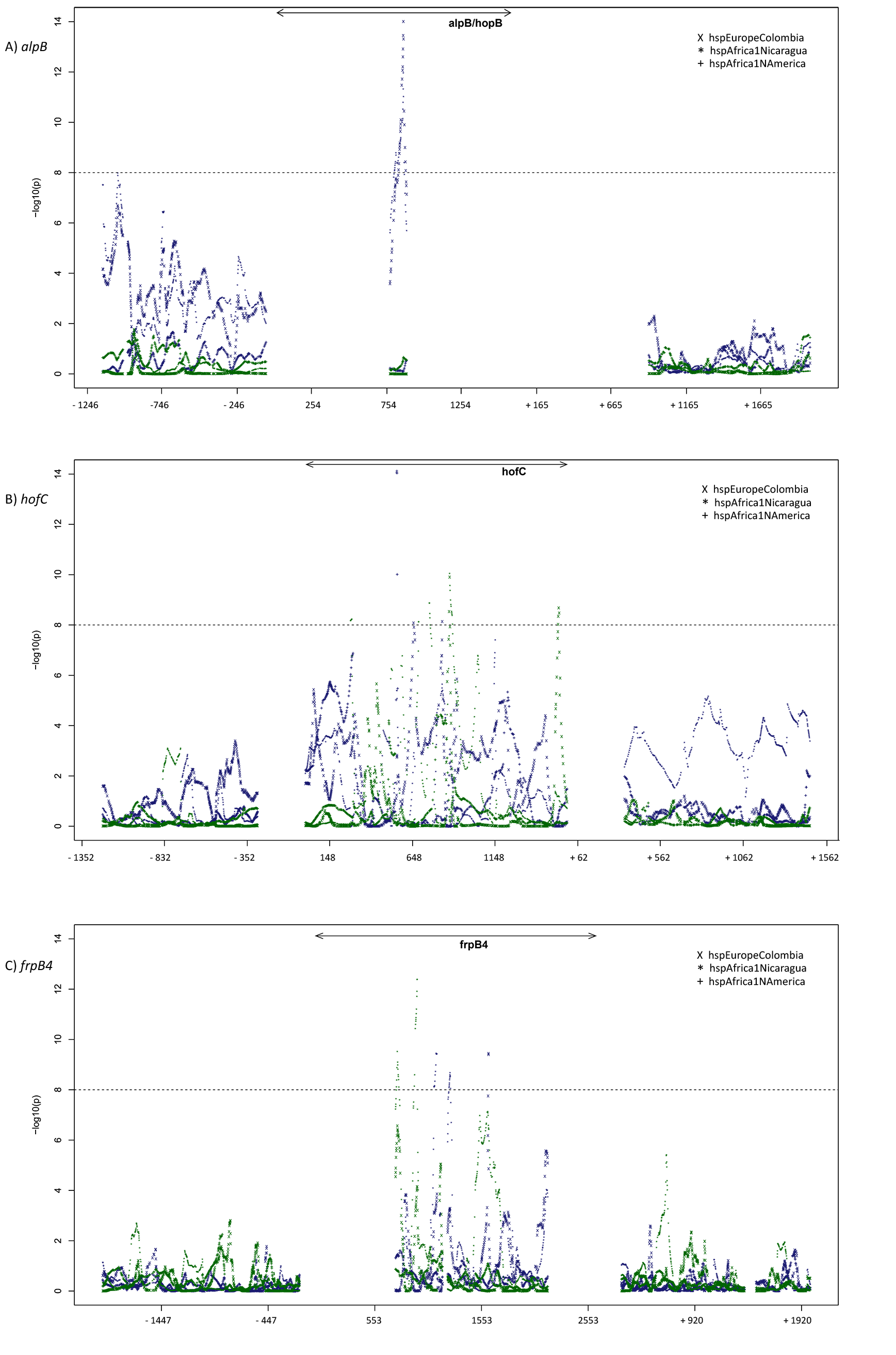

Supplement: S2 Fig — Each dot corresponds to a polymorphic site that was tested statistically. The three genes in Table 3 satisfying significance level p < 10−8 (p < 0.05 after Bonferroni correction) in more than one of the New World populations are shown. Blue symbols indicate the strength of statistical evidence for Asian enrichment and green European enrichment. Gaps represent sites where the missing frequency > 1% and sites in non-coding regions. A) alpB, B) hofC, and C) frpB4. (TIF) [file pgen.1006546.s002.tif]

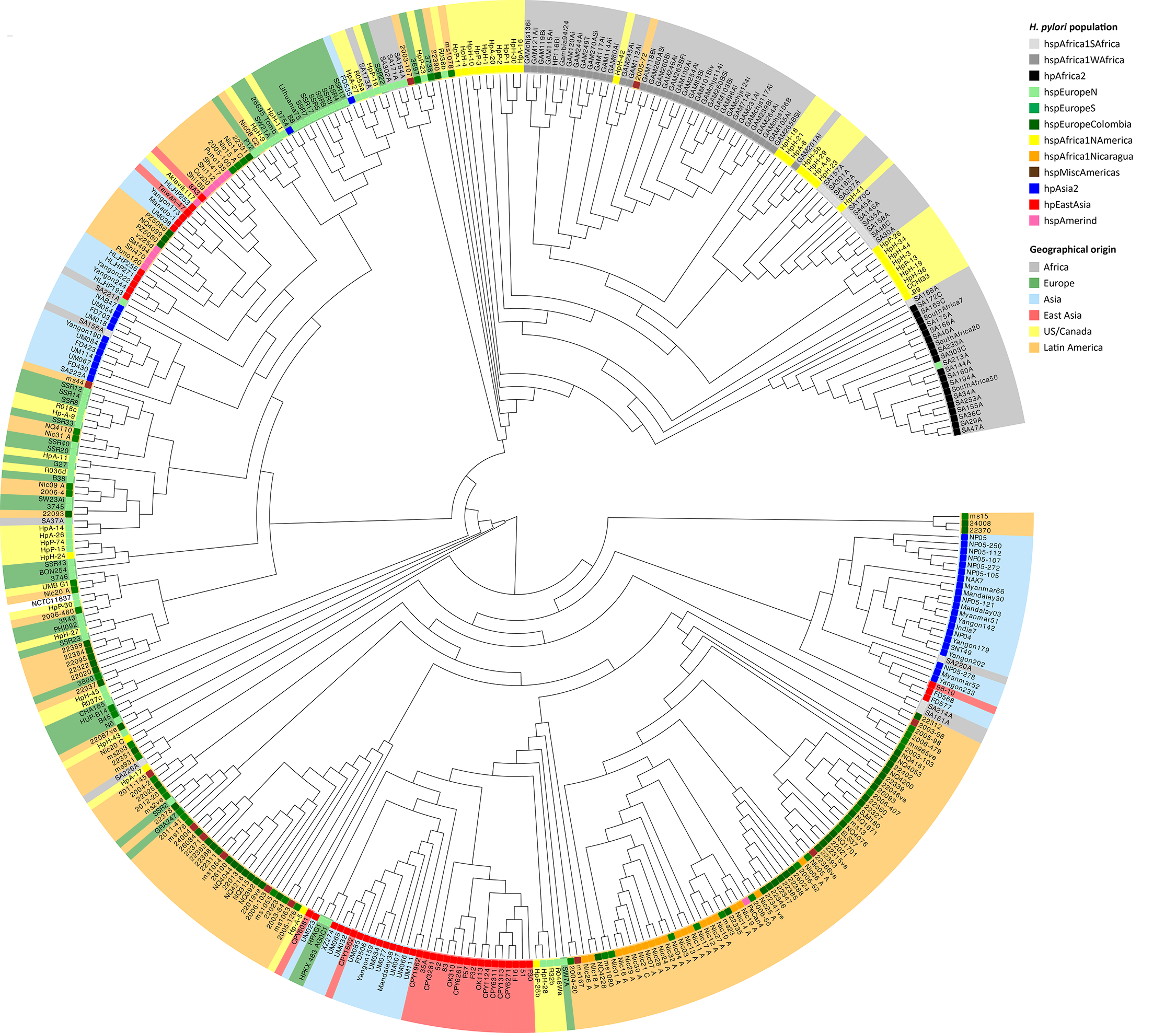

Supplement: S3 Fig — Leaves are shaded according to geographical origin and the H. pylori population assignment to according to the FineSTRUCTURE analysis is marked at the base of each leaf. (TIF) [file pgen.1006546.s003.tif]

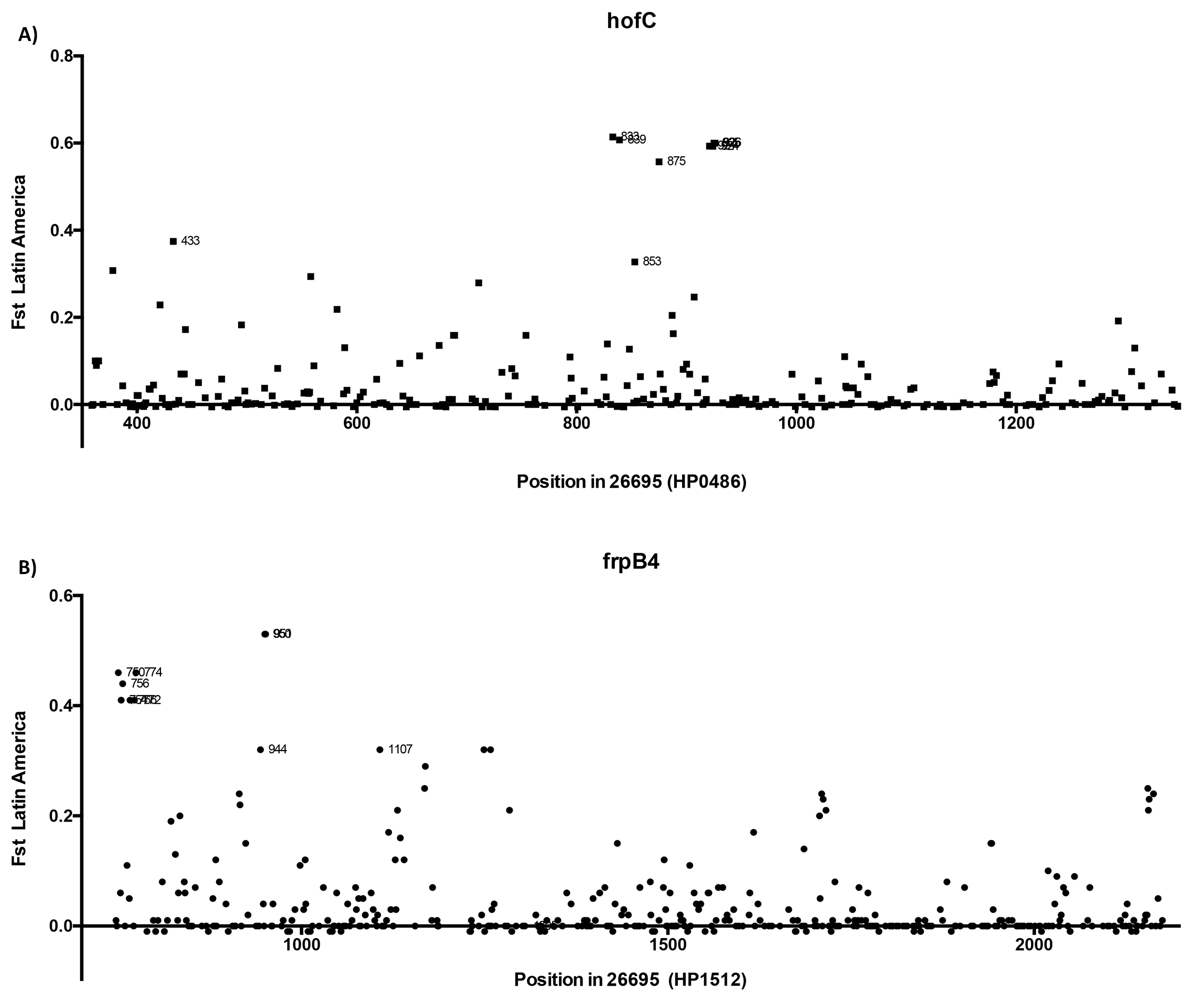

Supplement: S4 Fig — Each dot represents a nucleotide position. For positions with Fst > 0.25 the nucleotide position in 26695 is denoted. (TIF) [file pgen.1006546.s004.tif]

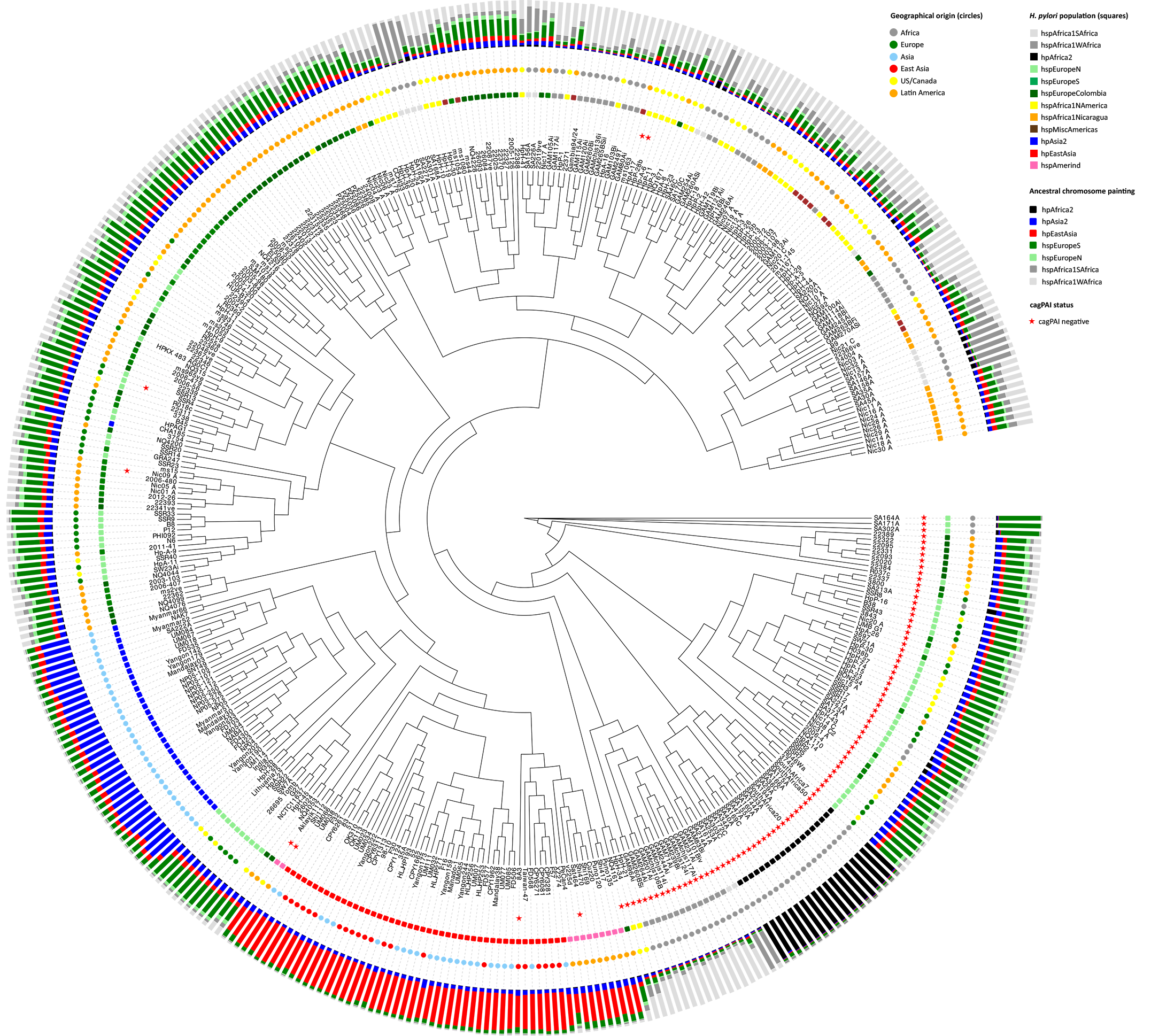

Supplement: S5 Fig — Neighbour-joining tree based on gene sharing distance (absence and presence of genes). The outer circle shows the Old World chromosome painting as in Fig 2A. Circles denote geographical origin and squares the H. pylori population assignment according to the FineSTRUCTURE analysis. Red stars are marking strains without the Cag Pathogenicity Island (CagPAI) (TIF) [file pgen.1006546.s005.tif]
